# Lung Macrophages Contribute to House Dust Mite Driven Airway Remodeling via HIF-1α

**DOI:** 10.1371/journal.pone.0069246

**Published:** 2013-07-23

**Authors:** Adam J. Byrne, Carla P. Jones, Kate Gowers, Sara M. Rankin, Clare M. Lloyd

**Affiliations:** 1 Leukocyte Biology Section, National Heart & Lung Institute, Imperial College London, London, United Kingdom; 2 Nuffield Department of Orthopaedics, Rheumatology and Musculoskeletal Sciences, Kennedy Institute of Rheumatology, University of Oxford, London, United Kingdom; University Hospital Freiburg, Germany

## Abstract

HIF-1α is a transcription factor that is activated during hypoxia and inflammation and is a key regulator of angiogenesis *in vivo*. During the development of asthma, peribronchial angiogenesis is induced in response to aeroallergens and is thought to be an important feature of sustained chronic allergic inflammation. Recently, elevated HIF-1α levels have been demonstrated in both the lung tissue and bronchoalveolar lavage of allergic patients, respectively. Therefore, we investigated the role of HIF-1α on the development of angiogenesis and inflammation following acute and chronic allergen exposure. Our data shows that intranasal exposure to house dust mite (HDM) increases the expression of HIF-1α in the lung, whilst reducing the expression of the HIF-1α negative regulators, PHD1 and PHD3. Blockade of HIF-1α *in vivo*, significantly decreased allergic inflammation and eosinophilia induced by allergen, due to a reduction in the levels of IL-5 and Eotaxin-2. Importantly, HIF-1α blockade significantly decreased levels of VEGF-A and CXCL1 in the lungs, which in turn led to a profound decrease in the recruitment of endothelial progenitor cells and a reduction of peribronchial angiogenesis. Furthermore, HDM or IL-4 treatment of primary lung macrophages resulted in significant production of both VEGF-A and CXCL1; inhibition of HIF-1α activity abrogated the production of these factors via an up-regulation of PHD1 and PHD3. These findings suggest that novel strategies to reduce the expression and activation of HIF-1α in lung macrophages may be used to attenuate allergen-induced airway inflammation and angiogenesis through the modulation of VEGF-A and CXCL1 expression.

**Clinical Relevance:**

This study provides new insights into the role of HIF-1α in the development of peribronchial angiogenesis and inflammation in a murine model of allergic airway disease. These findings indicate that strategies to reduce activation of macrophage derived HIF-1α may be used as a target to improve asthma pathology.

## Introduction

Asthma is a heterogeneous chronic inflammatory disease of the airways, characterized by airway hyperresponsiveness, eosinophilic inflammation and is associated with airway remodeling. Airway remodeling consists of structural changes in the lungs typified by deposition of extracellular matrix proteins, mucus hypersecretion, and airway smooth muscle hypertrophy and importantly, the formation of new peribronchial blood vessels. The formation of new blood vessels (named angiogenesis) is believed to be necessary to sustain chronic inflammation [1], [2]. The most numerous immune cell-type present in the non-diseased lung, macrophages are ideally placed to influence pulmonary inflammation, airway remodeling and angiogenesis. Macrophages have been shown to be a major source of pro-angiogenic factors, in both human and mouse studies [3], [4]. However, the role of macrophages in HDM-driven angiogenesis is not well understood.

Hypoxia-inducible factor 1 (HIF-1) belongs to a family of proteins that regulate the cellular response to oxygen deficit (hypoxia) in order to minimize tissue damage. HIF-1 is a heterodimeric transcription factor composed of an oxygen sensitive HIF-1α subunit and a constitutively expressed HIF-1β subunit [5], [6]. HIF-1 is primarily regulated at the protein level by hydroxylation by a family of prolyl hydroxylase domain-containing enzymes (PHD), leading to its ubiquitination and degradation in proteasomes. To date, 4 PHD enzymes have been described that regulate HIF-1 activity *in vivo* (PHD1, PHD2, PHD3 and P4H-TM) [5], [6]. Several studies have shown that silencing expression of PHDs *in vivo* induces up-regulation of HIF-1α and consequently leads to an increase in angiogenesis in response to ischemic injury [7]. However, the contribution of these enzymes during angiogenesis induced by allergen is yet to be established.

Activation and induction of gene transcription by HIF-1 is normally associated with hypoxia, however it is now well established that HIF-1α can also be activated in a normoxic environment during an inflammatory response by hypoxia-independent signals such as transforming growth factor-β, LPS, TNF-α and GM-CSF [8]–[10]. Recently, studies have suggested that HIF-1α is correlated with the development of allergic airway inflammation induced by ovalbumin in murine models [11]–[17]. Moreover, HIF-1α levels are increased in lung tissue and bronchial fluid of patients with asthma and in the nasal fluid of patients with rhinitis after allergen challenge [17]. Interestingly, depletion of the constitutive subunit, HIF-1β, also results in diminished allergic inflammation, suggesting that the entire HIF-1 complex and not just the HIF-1α subunit, is necessary in the development of allergic inflammation [18]. One of the most important HIF-1α target genes is vascular endothelial growth factor A (VEGF-A). VEGF-A is a potent pro-angiogenic factor and its levels have been shown to be increased in the bronchoalveolar lavage from asthmatic patients [19], [20]. Furthermore, VEGF-A has been demonstrated to be a stimulator of inflammation, airway remodeling, and physiologic dysregulation in murine models of allergic airway diseases [21]. These data highlight the potential importance of HIF-1 and VEGF-A during allergy pathology; but their direct role in mediating allergic pathology has not been investigated.

Bone marrow-derived endothelial progenitor cells (EPCs) can differentiate into mature endothelial cells in adult animals, including humans. These cells play an important role in angiogenesis in a variety of physiological and pathological processes by integrating into new blood vessels and by secreting different pro-angiogenic factors *in situ* [22]. Asthmatic patients present elevated numbers of circulating EPCs [23]. In addition, we and others have shown that EPCs are rapidly recruited to the lungs after ovalbumin allergen challenge [24], [25]. Moreover, Jiang and collaborators have shown that over expression of HIF-1α in EPCs leads to increased mobilization, recruitment and function of these cells after ischemic injury, resulting in increased angiogenesis [25], [26]. However, to date there is no study to our knowledge that shows the effects of HIF-1 inactivation in EPC recruitment during the allergic response.

Since HIF-1α has a key role in the production of pro-angiogenic factors and in EPC function, we evaluated whether blockade of HIF-1α activation *in vivo* could alter EPC recruitment to the lungs, angiogenesis and inflammation induced by the clinically relevant allergen, house dust mite. We determined that HIF-1α expression is increased in the lung tissue after acute and chronic exposure to allergen, whilst the expression of both PHD1 and PHD3 are decreased. We show that HIF-1α plays a central role in the development of allergic inflammation induced by house dust mite. Blockade of HIF-1α during HDM-induced airway inflammation resulted in a profound decrease in the recruitment of endothelial progenitor cells to the lungs with subsequent reduction of peribronchial angiogenesis. HIF-1α was found to be localized in mononuclear cells in the submucosa of lung tissue and the observed alterations in the angiogenic potential of the lung were attributed to HIF-1α-expressing macrophages. Direct stimulation of murine primary macrophages *ex vivo* with either HDM or IL-4, resulted in enhanced production of CXCL1 and VEGF, with a concomitant inhibition of PHD1 and 3. Furthermore, HIF-1α blockade inhibited macrophage derived CXCL1 and VEGF production via up-regulation of both PHD1 and PHD3. These findings identify a key role for HIF-1α pathway in the innate response of macrophages to inhaled aero-allergen and furthermore, that new strategies to reduce expression and/or activation of HIF-1α in lung macrophages may be a novel strategy to attenuate allergen-induced airway inflammation and pulmonary angiogenesis.

## Materials and Methods

### Reagents

General laboratory reagents were purchased from either Life Technologies (Paisley, UK) or Sigma-Aldrich (Poole, UK). Chetomin (CTM) was purchased from Enzo Life Science UK LTD (Exeter, UK).

### Induction and Analysis of Allergic Airway Inflammation

Female BALB/c mice 6–8 weeks old (Charles River, Morgate, UK) received 15 µg HDM extract (*Dermatophagoides pteronyssinus* in phosphate-buffered saline, PBS) (Greer Laboratories, Lenoir, NC) or 15 µl PBS intranasally 3 days a week for 1, 3 or 5 weeks. In blocking experiments, mice were given chetomin (3 mg/mL) intraperitoneally (i.p.) 2 hours before each intranasal challenge with either PBS or HDM. UK Home Office guidelines for animal welfare based on the Animals (scientific procedures) act 1986 were strictly observed. The protocol was approved by the Imperial College London Animal Welfare and Ethical Review Body (AWERB). All surgery was performed under ketamine and sodium pentobarbital anaesthesia, and all efforts were made to minimize suffering.

### Measurement of AHR

AHR was determined by direct measurements of resistance in anesthetized and tracheostomized mice in response to inhaled methacholine (MCh; Sigma, Cambridge, UK) at concentrations of 3 to 100 mg/ml for 1 minute in an EMMS system (EMMS, Hampshire, UK), [24].

### Sample Preparation

Serum, BAL fluid, lung homogenates were harvested as previously described [27]. Eosinophils in lung digests were analysed by flow Cytometry as Siglec positive and GR1 intermediate granulocytes.

### Immunohistochemistry

Lung paraffin sections (5 µm) were stained with rabbit anti-human Von Willebrand Factor (vWF) (1∶200, A0082, Dako UK Ltd) to identify blood vessels. This antibody has been shown to cross-react with murine vWF [28]. The number of peribronchial vessels per airway was counted in at least 4 airways per section. Vessels were scored if no more than 50 µm from the basal membrane.

### Endothelial Progenitor Cell Assay

Mice were sacrificed under terminal anaesthesia at 24 hours after the last HDM exposure. In preliminary experiments, 1 mL of PBS was flushed through the heart to eliminate blood contamination in the lung. The biggest lobe from the lungs were minced, and digested with 0.15 mg/ml collagenase type D, and 25 µg/ml DNase type I (30 min, 37°C). Cell suspensions were prepared by filtration through a Cell Strainer (Falcon 2360, 100 µm, nylon) and separated on a discontinuous Percoll gradient. 10^6^ cells were plated in EBM-2 media supplemented with VEGF (50 ng/ml) and 17% FCS (Cambrex BioScience Walkersville, Inc.) on a fibronectin (10 µg/mL)-coated dish. In selected experiments, bone marrow was collected and 10^6^ cells plated using the same method used for lungs. EPC colonies were scored at day 21 on an inverted microscope by morphology as described previously [28].

#### Chemokine analysis

Chemokine levels were measured in lung homogenates (50 mg/ml). Paired antibodies for murine CXCL1, SDF-1, Eotaxin 1, Eotaxin 2 and VEGF (R&D systems, Abingdon, UK) were used in standardized sandwich ELISAs according to the manufacturer’s protocol.

#### Macrophage culture

Cells from lung and BAL fluid were collected as before. In order to isolate primary macrophages, cells obtained from lung digests were left to adhere in plastic dishes for 2 hours (10^5^ cells/mL). Cells were washed twice with saline and incubated with RPMI 10% FCS with HDM (5 ug/mL) or 10 ng/mL recombinant murine IL-4 (PeproTech) with or without different concentrations of HIF-1α inhibitor (chetomin). Supernatants were collected for measurement of cytokines at the indicated timepoints.

#### Statistical analysis

Data were analyzed using Prism 4 for Windows from GraphPad Software Inc, using Kruskal-Wallis or Mann-Whitney tests.

## Results

### Acute and Prolonged HDM Challenge Leads to an Increase in the Levels of HIF-1α in the Lung

**Figure 1:**
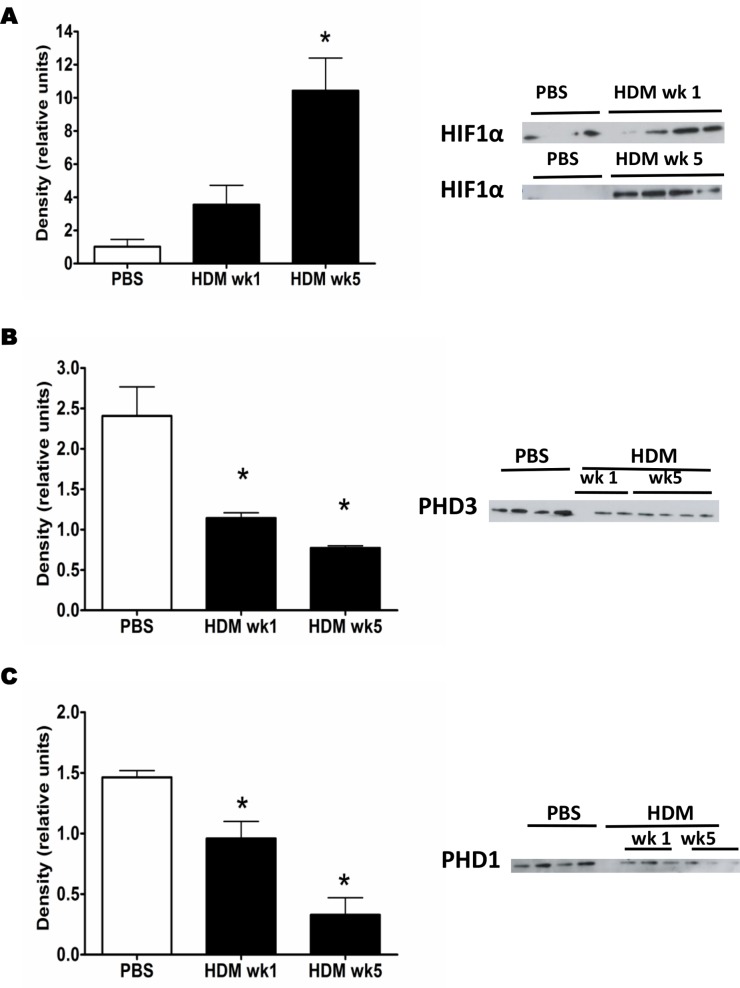
HDM challenge induces HIF-1α regulation. Western blot analysis of HIF-1α (A), PHD3 (B) and PHD1 (C) in lung tissue homogenates of mice treated with PBS or HDM three times a week for 1 or 5 weeks. Bars indicate mean ± SEM of the density of the Western blot bands. *represents *p*<0.05 compared to PBS controls. Data derived from 4 mice per time point per group.

There is growing evidence that hypoxia-inducible transcription factors are involved in the pathophysiology of asthma. Hypoxia-inducible factor-1α (HIF-1α) regulates the expression of many hypoxia regulated genes, including key genes involved in angiogenesis such as the gene that encodes VEGF-A [29]. However, the direct contribution of HIF-1α activation during the angiogenic response to allergen is not yet fully understood. We therefore first sought to evaluate the expression of HIF-1α after mice were exposed to inhaled HDM for different periods of time. Our data shows an early increase in the expression of HIF-1α in the lungs after 1 and 5 weeks of allergen challenge (Figure 1A). HIF-1α expression is regulated by proteolysis, following oxygen-dependent hydroxylation of specific prolyl residues, by enzymes named prolylhydroxylase (PHD) 1, 2 and 3. Expression of the endogenous HIF-1α-regulators, PHD3 and PHD1 (but not PHD2), was substantially reduced after HDM challenge (Figure 1C, D and Figure S1, respectively) in comparison to the PBS group.

### Blockade of HIF-1α or VEGF-A Inhibits Allergic Inflammation in vivo

**Figure 2:**
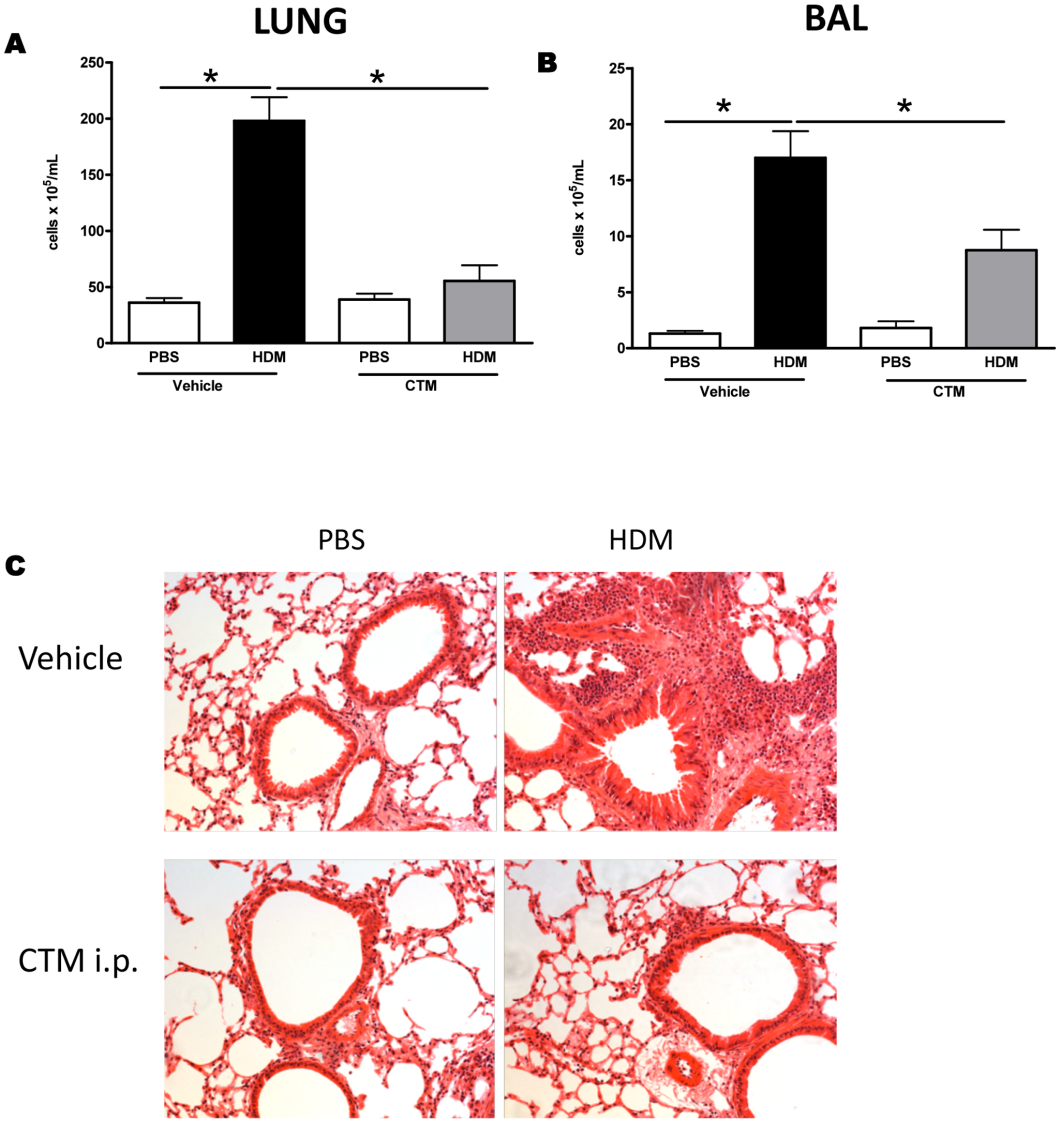
Blockade of HIF-1α or VEGF-A inhibits allergic inflammation *in vivo*. Mice were treated with PBS or HDM for 5 weeks, with vehicle or chetomin, administered intraperitoneally 20 minutes prior to each allergen challenge. Total cell numbers in the lungs (A) and BALF 24 hours after last allergen challenge (B). Representative photomicrographs of lungs stained with haematoxylin and eosin (panel C). *represents *p*<0.05. n = 4–8 mice per experimental group.

**Figure 3:**
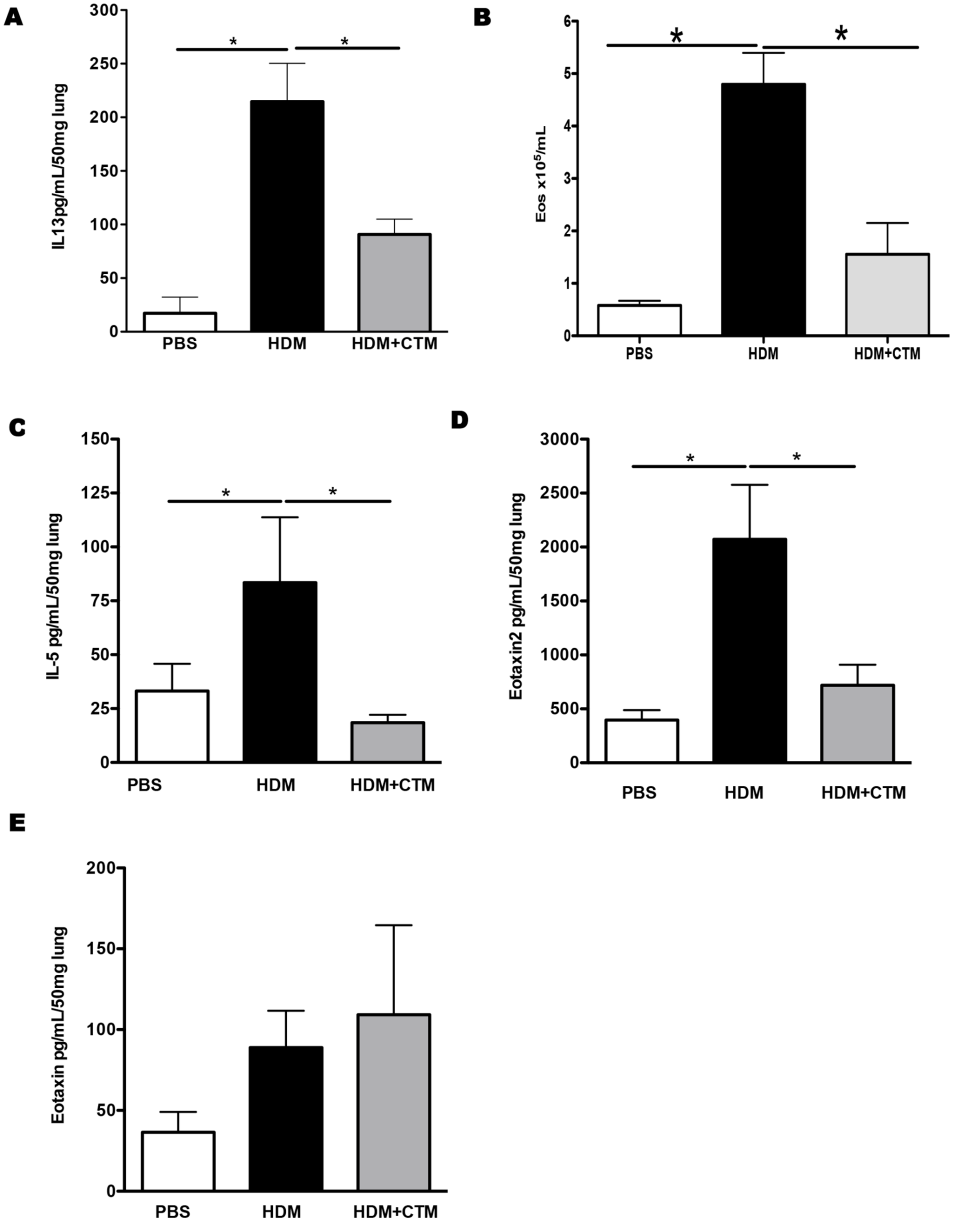
Blockade of HIF-1α activity *in vivo* reduces lung eosinophilia induced by HDM exposure. Mice were treated with PBS or HDM intranasally for 5 weeks, and chetomin was administrated intraperitoneally 20 minutes prior to each challenge. IL-13 levels were measured by ELISA in lung tissue (panel A). Mean ± SEM number of eosinophils in the lung (panel B). IL-5 (panel C), eotaxin 2 (panel D) and eotaxin 2 (panel E) were measured in the lung tissue homogenates by ELISA. *represents *p*<0.05. n = 4–8 mice per experimental group.

To further assess the role of HIF-1α in the development of the allergic response, we evaluated the effect of HIF-1α blockade *in vivo*. In order to modulate the expression of HIF-1α we utilized Chetomin (CTM), a pharmacological inhibitor which has been demonstrated to attenuate hypoxia-inducible transcription by blocking the interaction of HIF-1α with transcriptional co-activators p300 and cAMP response element binding (CREB) protein [30]. BALB/c mice were exposed to HDM for 5 weeks and HIF-1α activity was blocked by administration of CTM, i.p., 20 minutes before each airway challenge. Exposure of mice to inhaled HDM resulted in changes in lung function and recruitment of inflammatory leukocytes as observed previously. HDM-induced inflammation was significantly reduced by HIF-1α blockade in both lung tissue and in the bronchoalveolar lavage (Figure 2A, B and C). Chronic exposure of mice to HDM leads to the development of increased airway hyperreactivity to methacholine, but this is not modulated after inhibition of HIF-1α (Figure S2). In contrast, levels of IL-13 were decreased in the lung following HIF-1α blockade (Figure 3A). Further analysis of inflammation revealed that the characteristic eosinophil recruitment induced by exposure to HDM is abrogated in the absence of HIF-1α activity Figure 3B. Moreover, blockade of HIF-1α led to a significant reduction in the levels of the eosinophil growth and survival factor, IL-5 and the chemoattractant Eotaxin 2 in the lungs (Figures 3C and 3D). In contrast levels of Eotaxin-1 remained unchanged following HIF-1α blockade (Figure 3E).

### Blockage of HIF-1α Reduces Production of Pro-angiogenic Factors, Endothelial Progenitor Cell Recruitment and Angiogenesis

**Figure 4:**
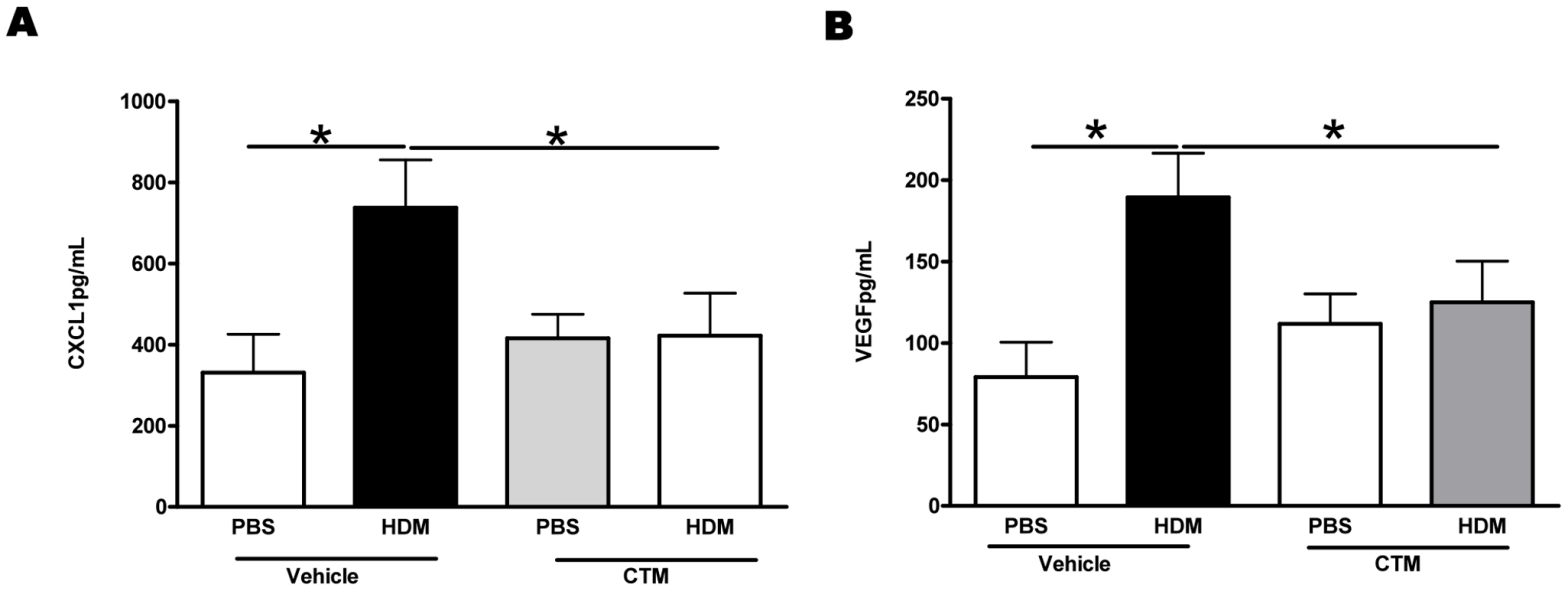
Blockade of HIF-1α *in vivo* reduces the production of pro-angiogenic mediators in the lung. CXCL1 (A) and VEGF-A (B) levels were measured in the lung tissue homogenates by ELISA. *represents *p*<0.05. n = 4–8 mice per experimental group.

**Figure 5:**
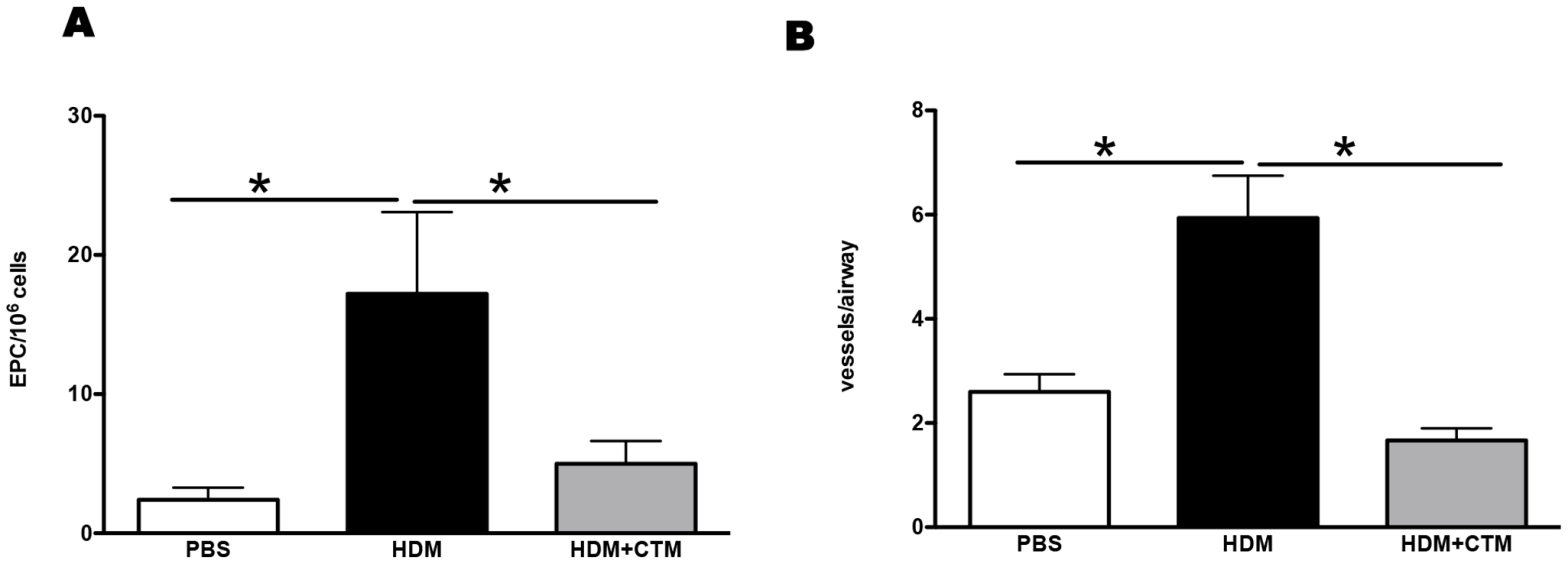
Blockade of HIF-1α *in vivo* modulates angiogenesis and EPC recruitment to the lungs. Mice were treated with PBS or HDM intranasally three times a week for 1 week, and chetomin administered 20 minutes prior to each challenge. (A) Data represents mean ± SEM of EPCs per 10^6^ lung mononuclear cells enumerated after 21 days of culture, as described in methods. (B) Data represents mean ± SEM of peribronchial blood vessels per square millimetre. *represents *p*<0.05. n = 4–8 mice per group.

Mice were exposed to HDM or PBS for 1 week and treated with HIF-1α inhibitor or vehicle, 2 hours before each allergen challenge. At this early time point levels of CXCL1 and VEGF-A were significantly elevated in the lungs compared to PBS controls (Figure 4A and B). However, those mice that had received the HIF-1α inhibitor, exhibited levels of both CXCL1 and VEGF-A, which were comparable to those in the PBS-treated group. Blockade of HIF-1α significantly reduced the recruitment of endothelial progenitor cells to the lung in response to HDM (Figure 5A and Figure S3). Furthermore, blockade of HIF-1α lead to a complete inhibition of the angiogenic response to allergen challenge, since the HDM induced increase in vessels per airway was completely abrogated in the absence of HIF-1α signalling (Figure 5B).

These data suggest that the pulmonary production of VEGF-A and CXCL1 in response to allergen is dependent on the activation of HIF-1α.

### HIF-1α Blockade Inhibits the Production of VEGF-A, CXCL1 and Eotaxin-2 in Lung Macrophages

**Figure 6:**
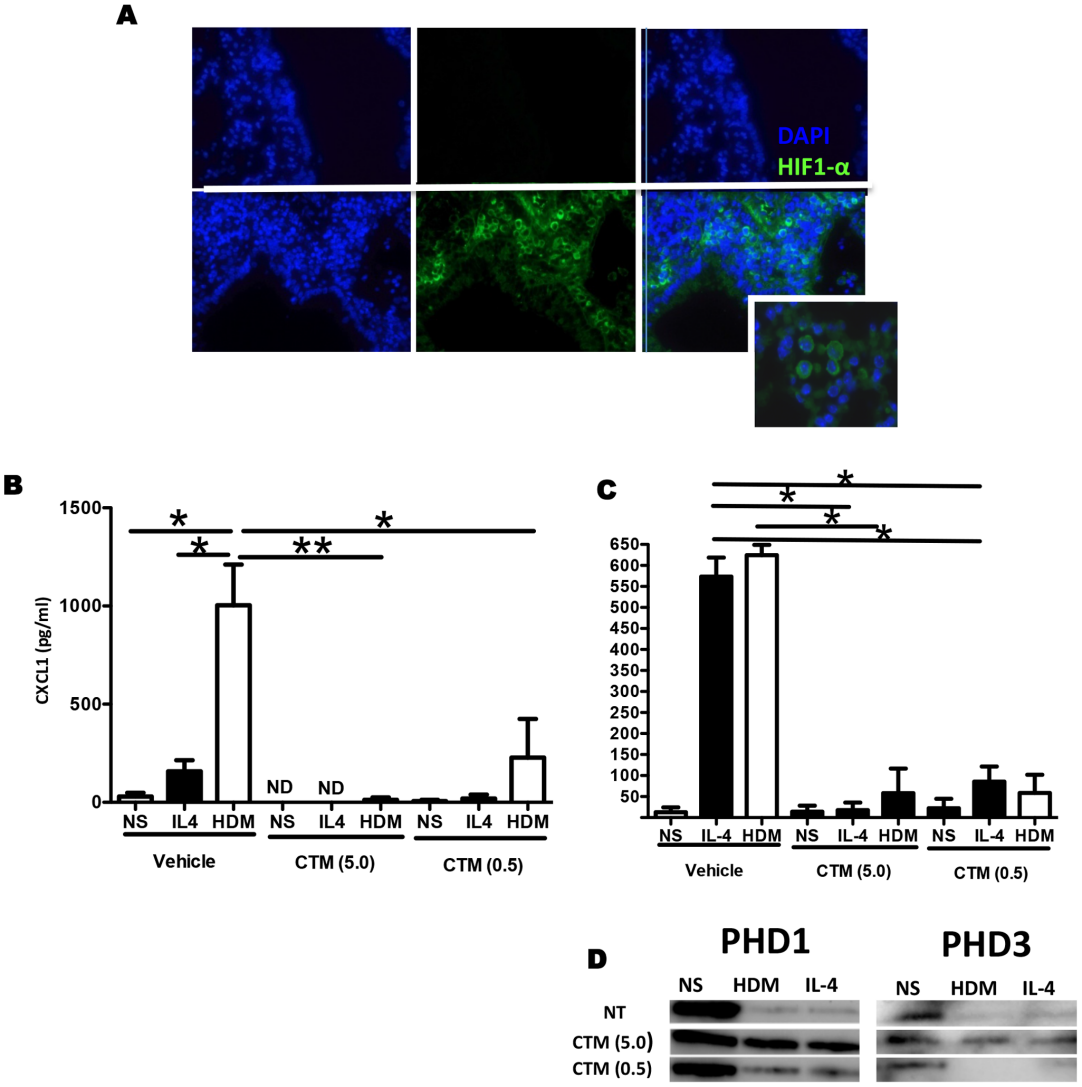
HDM induced upregulation of VEGF-A and CXCL1 in macrophages is HIF-1α dependent. A. Representative photomicrographs of lungs from mice treated with PBS (top panel) or HDM (bottom panel) for 1 week, immunofluorescently stained for HIF-1α (green) and counter stained with DAPI (blue). Original magnification 400×. Isolated macrophages from lung tissue and were stimulated with HDM *in vitro*, for 24 h, in the presence or absence of HIF-1α blockade. CXCL1 (A) and VEGF-A levels were measured by ELISA in the culture supernatants. Expression of PHD1 and PHD3 (D) was assessed by western blotting of cell lysates. Bars represent mean ± SEM. Data derived from 3 independent experiments. *represents *p*<0.05, **represents *p*<0.01.

Since HDM challenge induced an increase in HIF-1α expression, we evaluated the localization of this transcription factor in lung sections from HDM or PBS challenged mice. Figure 6A shows that HIF-1α expression is mostly detected in mononuclear cells in the submucosa of lung tissue from HDM allergic mice. As our data indicate that blockade of HIF-1α signalling leads to diminished production of the pro-angiogenic mediators VEGF-A and CXCL1 (Figure 4A and B), and that these mediators were likely derived from lung macrophages, we next isolated macrophages from lung tissue to explore their function *ex vivo*. Exposure of primary tissue macrophages to HDM was sufficient to induce the production of CXCL1 and VEGF (Figure 6B and C). Interestingly, HIF-1α activation is required for the production of these factors, since blockade of HIF-1α activity *in vitro* with chetomin (CTM) significantly reduced production of CXCL1 and VEGF-A by these cells (Figure 6B and C). Furthermore, the inhibitory effect observed was associated with decreased expression of the HIF-1α regulatory factors PHD1 and PHD3 (Figure 6D). Interestingly, rIL-4 treatment of primary lung macrophages resulted in release of CXCL1 and VEGF release from these cells, at levels which were comparable to those found in HDM treated cultures (Figure 6B and C). The effects of IL-4 on primary macrophages were inhibited by chetomin, suggesting that HIF-1α blockade may be useful not only specifically in the context of HDM exposure, but also in the setting of Th2 driven processes. Taken together, these data indicate that activation of macrophages derived HIF-1α is sufficient to elicit the production of pro-angiogenic factors and to promote pulmonary angiogenesis in response to HDM challenge.

## Discussion

In this study we have investigated the role of HIF-1α in a model of allergic airways disease. Macrophage derived-HIF-1α was found to be critical in promoting inflammation and angiogenesis in response to allergen challenge, including the production of pro-angiogenic factors and recruitment of endothelial progenitor cells (EPCs) to the lungs. This is the first time that this transcription factor has been implicated in EPC recruitment and neovascularisation during allergic inflammation *in vivo*.

Neovascularization plays a well-known role in inflammation and tissue remodeling in several chronic inflammatory disorders, including asthma. Biopsies from asthmatic patients show an increase in both blood vessel number and size of vessels, and these changes are associated with vascular leakage [31]–[33]. Changes in vascularity correlate with disease severity, possibly due to a contribution to airflow limitation in asthmatic patients. Several studies have also shown that asthmatic patients exhibit higher levels of VEGF-A and other angiogenic factors in BAL fluid and serum [32]. In the present study we evaluated formation of new blood vessels using a murine model of inhaled chronic allergen challenge using a common and clinically relevant aeroallergen, namely house dust mite (HDM). This model reproduces the classic features of asthma including inflammation, increase in collagen deposition, mucus production and airways smooth muscle cell proliferation [34]. We show that HDM challenge leads to an increase in peribronchial blood vessels, similar to that observed in biopsies from asthmatic patients. Moreover, we show that the angiogenic switch in response to HDM is an early event that precedes the other airway remodeling features. This is important since increased airway vascularity has been found even in childhood asthma [35]–[37]. However, the mechanism underlying these early changes in vascularity is not well understood.

Endothelial progenitor cells (EPC) are thought to facilitate the development of new blood vessels in both developmental and adult life [22]. Previously, we have shown that ovalbumin challenge in sensitized mice leads to recruitment of bone-marrow derived EPCs due to an increase in the levels of CXCL1 in the lungs [25]. In the current study we show a similar effect using an inhaled allergen, with HDM exposure leading to significant EPC recruitment to the lungs. Trafficking of EPCs to the lungs requires their mobilization from the bone-marrow into the blood and subsequent recruitment from the blood to the inflamed tissue. HDM challenge also results in increased pulmonary levels of CXCL1, the chemokine that is necessary for recruitment of EPC to the lungs. Interestingly we also show that HDM exposure leads to an increase in EPC numbers in the bone-marrow, suggesting that HDM promotes an increase in the pool of these progenitor cells that can be mobilized during chronic inflammation.

Hypoxia inducible factor-1α (HIF-1α) is a transcription factor that is activated in response to low levels of oxygen in order to minimize tissue damage. However hypoxia independent signals can also trigger HIF-1α activation in a normoxic environment [8]–[10]. HIF-1α activation promotes the induction of several pro-angiogenic genes, including VEGF-A. Recently, HIF-1α has been demonstrated to be responsible for LPS-induced IL-1β expression in bone marrow derived macrophages [38]; furthermore, HIF-1α activation has been shown to be correlated with chronic diseases such as asthma [39]. Previously HIF-1α expression was detected in epithelial cells after ovalbumin challenge in sensitized mice [15]. However, Lee *et al* have shown that asthmatic patients exhibit increased numbers of HIF-1α positive cells in the submucosa of bronchial biopsy compared to control subjects [17]. Expression of HIF-1α is regulated by the PHD (prolyl hydroxylases) enzymes which act by catalyzing the hydroxylation of proline residues in the HIF-1α molecule, directly affecting its degradation in the proteasomes. It is well established that PHDs are important in regulation of HIF; however, little is known about their role during inflammatory responses. Although the three PHDs are capable of regulating HIF-1α, their activity and cellular distribution varies, suggesting that their role in the angiogenic response might also differ. Indeed, Walmesley *et al* have shown that PHD3 is a selective regulator of neutrophil hypoxic survival [40]. In this study the authors have also shown that PHD3 and PHD2 levels, but not PHD1, are increased in circulating neutrophils from individuals with rheumatoid arthritis compared to normal subjects. Interestingly, our data shows for the first time that even though all 3 PHDs are detected in murine lungs, only the expression of PHD1 and PHD3 can be modulated by allergen exposure. Taking together these data highlight the importance of a better understanding on the tissue and disease specificity of these enzymes and suggest that targeting PHD1 and PHD3 for therapeutic purpose in allergic diseases would be more efficient than PHD2.

In order to determine the functional consequence of HIF-1α suppression *in vivo* we used the pharmacological inhibitor chetomin to block the HIF-1α pathway during HDM challenge. Cell recruitment to the lungs and airways was reduced in mice given chetomin before HDM challenge. In particular, accumulation of eosinophils was reduced almost to baseline levels. Interestingly, previous studies have shown that heterozygous-null mice in HIF-1α are protected from lung eosinophilia [41]. Moreover, an earlier study also showed that blockade of HIF-1α expression during acute ovalbumin challenge leads to decreased inflammation [14]–[16]. In our study, Chetomin induced HIF-1α blockade abrogated secretion of TH2 cytokines IL-5 and IL-13, and eotaxin 2 in the lung, but not eotaxin 1. Eotaxin 1 can be expressed by epithelial cells, while the other mediators are produced mainly by cells in the submucosa, suggesting that the epithelial response to HDM might be unaffected by HIF-1α activity. In contrast, Kim *et al* concluded that amelioration of allergic inflammation via HIF-1α blockade occurred by suppression of VEGF in bronchial epithelial cells [12]. However, these dissimilarities likely reflect the different inhibitors and models used – particularly since our study utilised an inhaled allergen challenge protocol.

VEGF-A is up-regulated in response to allergen challenge in mice and asthmatic patients [42]–[44]. We found that blocking VEGF-A activity *in vivo* lead to a decrease in HDM induced inflammation similar to that observed after blocking HIF-1α activity. Lee *at al* have shown that over expression of VEGF-A in the lung epithelium leads to an increase in blood vessels, airway remodeling and TH2-response [21], suggesting that VEGF-A might have a key role in the allergic response. Recruitment of EPCs to the lungs relies on CXCL1, rather than VEGF-A in ovalbumin sensitized mice. Blockade of the CXCL1-CXCR2 axis specifically reduced the recruitment of EPCs to the inflamed lungs but not their mobilization from the bone-marrow to the circulation, suggesting that other factors are involved in this process [25]. Here we show that administration of a HIF-1α antagonist prior to HDM exposure decreases the accumulation of EPC in the lungs. Moreover we show that blockade of HIF-1α leads inhibits the production of VEGF-A and CXCL1 in the lungs after allergen inhalation. We have observed that expression of HIF-1α induced by allergic inflammation in mice is mainly present in the submucosa compartment, particularly in mononuclear cells. In parallel, we determined that HDM and/or IL-4 can promote VEGF-A and CXCL1 secretion by lung macrophages in a HIF-1α dependent manner. These data suggests that HIF-1α has the potential to act as a key factor in the angiogenic switch that occurs in the lungs during the allergic response.

In conclusion this is the first study to describe a critical and novel role for lung macrophage derived HIF-1α in mediating the formation of new blood vessels, inflammation and recruitment of EPCs to the lungs in response to chronic exposure to a common aero-allergen. Understanding the molecular role of HIF-1α and PHDs in development of allergic inflammation could lead to novel therapeutic strategies to decrease inflammation and angiogenesis observed in asthmatic patients.

## Supporting Information

Figure S1 Western blot analysis of PHD2 in lung tissue homogenates from mice treated with PBS or HDM intranasally for 1 week. Bars represent mean ± SEM of the density of the Western blot band normalized to actin.
(TIF)

Figure S2 Mice were treated with PBS or HDM intranasally three times a week for 5 week, and CTM administered 20 minutes prior to each challenge. Data represents mean ± SEM Analysis of airway hyperreactivity to methacholine (MCh) as determined by resistance measurements in tracheotomized restrained animals. Increased airway resistance (RI) was measured in response to increasing doses of MCh. Data shown represent means ± SEM (*n* = 4–6)
(TIF)

Figure S3 Mice were treated with PBS or HDM intranasally three times a week for 1 week. Data represents mean ± SEM of EPCs per 10^6^ lung mononuclear cells enumerated after 21 days of culture, as described in methods. (D) Data represents mean ± SEM of peribronchial blood vessels per square millimetre. *Represents *p*<0.05. n = 4–6 mice per group.
(TIF)
