# Supplementary figures and images for: Lung Macrophages Contribute to House Dust Mite Driven Airway Remodeling via HIF-1α
Source: PLoS One. 2013 Jul 23;8(7):e69246. doi: 10.1371/journal.pone.0069246 (PMC3720585; doi:10.1371/journal.pone.0069246)

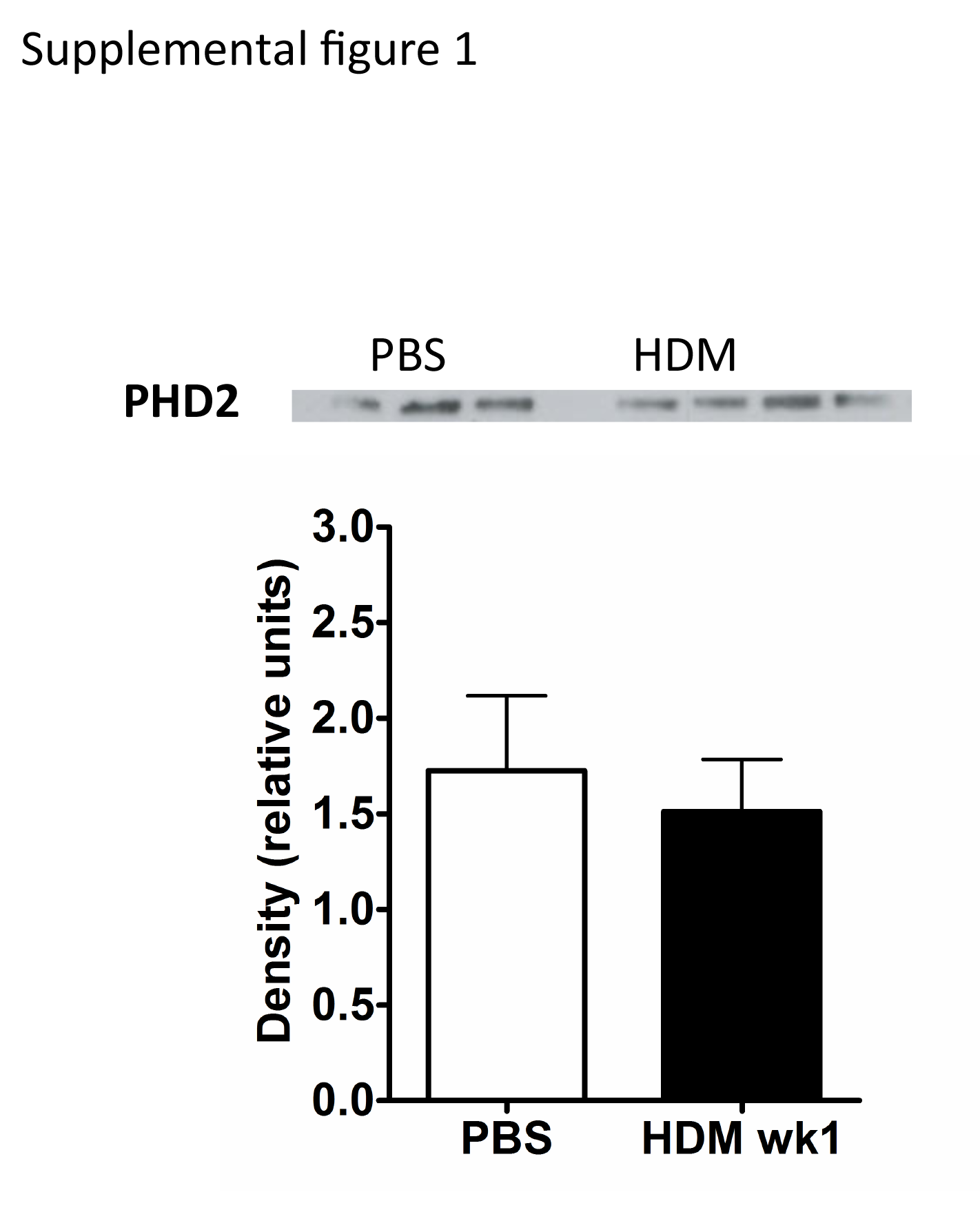

Supplement: Figure S1 — Western blot analysis of PHD2 in lung tissue homogenates from mice treated with PBS or HDM intranasally for 1 week. Bars represent mean ± SEM of the density of the Western blot band normalized to actin. (TIF) [file pone.0069246.s001.tif]

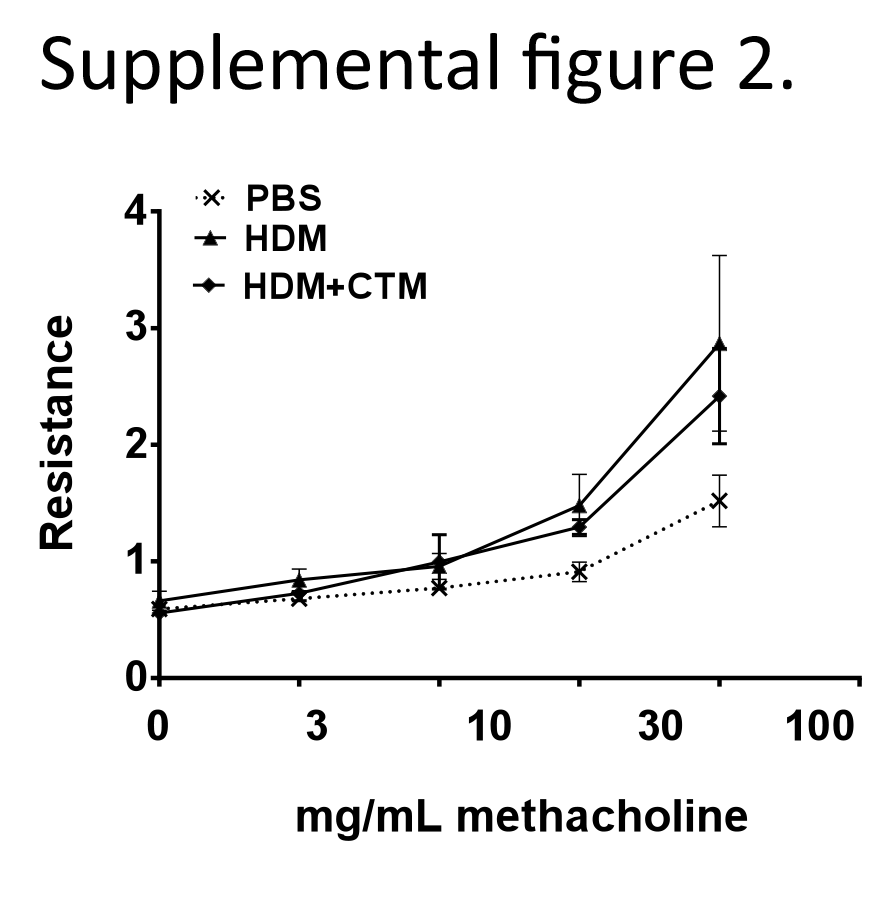

Supplement: Figure S2 — Mice were treated with PBS or HDM intranasally three times a week for 5 week, and CTM administered 20 minutes prior to each challenge. Data represents mean ± SEM Analysis of airway hyperreactivity to methacholine (MCh) as determined by resistance measurements in tracheotomized restrained animals. Increased airway resistance (RI) was measured in response to increasing doses of MCh. Data shown represent means ± SEM (n = 4–6) (TIF) [file pone.0069246.s002.tif]

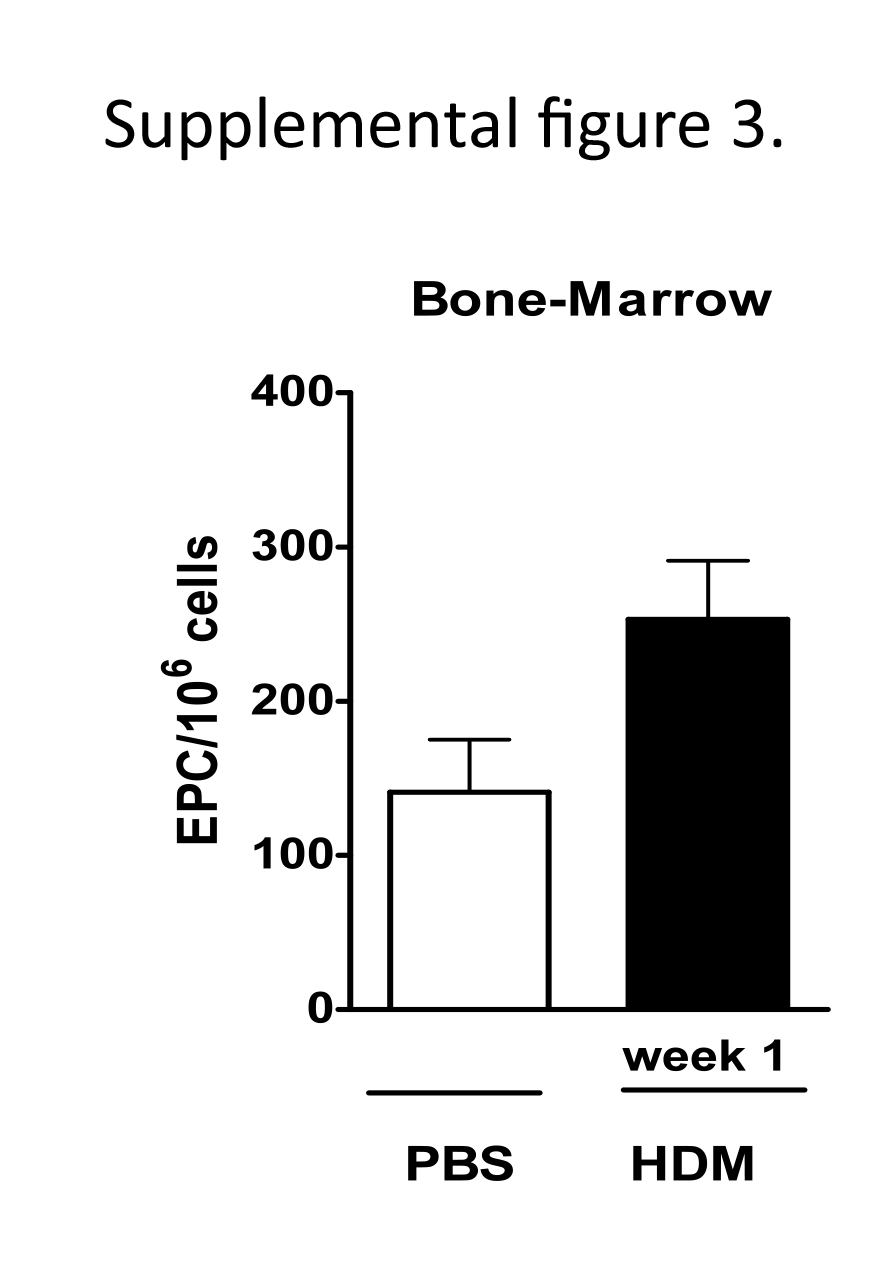

Supplement: Figure S3 — Mice were treated with PBS or HDM intranasally three times a week for 1 week. Data represents mean ± SEM of EPCs per 106 lung mononuclear cells enumerated after 21 days of culture, as described in methods. (D) Data represents mean ± SEM of peribronchial blood vessels per square millimetre. *Represents p<0.05. n = 4–6 mice per group. (TIF) [file pone.0069246.s003.tif]
